# Assessment of a Daily Diary Study Including Biospecimen Collections in a Sample of Sexual and Gender Minority Young Adults: Feasibility and Acceptability Study

**DOI:** 10.2196/52195

**Published:** 2024-02-19

**Authors:** Stephanie H Cook, Erica P Wood, Mariana Rodrigues, Janice Jachero Caldas, Maxline Delorme

**Affiliations:** 1 Department of Social and Behavioral Sciences School of Global Public Health New York University New York, NY United States; 2 Department of Biostatistics School of Global Public Health New York University New York, NY United States

**Keywords:** study protocol, young sexual minority men, YSSM, cardiovascular disease risk, health behaviors

## Abstract

**Background:**

Young sexual minority men (YSMM) engage in cardiometabolic risk behaviors (eg, substance use) at higher rates than their heterosexual counterparts. Theory and previous research suggest that these risk behaviors may stem, in part, from exposure to minority stress (ie, discrimination based on sexual identity and other identities such as race).

**Objective:**

This pilot study examined the feasibility and acceptability of a virtual 2-day daily diary study that examined daily experiences with discrimination, cardiometabolic risk behaviors (ie, sleep, physical activity, and substance use behaviors), and patterns of physiological stress and inflammation among YSMM aged 18 to 35 years.

**Methods:**

Participants (n=20) were recruited from the greater New York metropolitan area and engaged in a 2-day daily diary protocol wherein they provided web-based consent, took a web-based baseline survey, and then, starting the next day, provided 3 saliva samples a day for 2 consecutive days to measure salivary cortisol, engaged in 3 daily diaries per day, and provided 1 blood spot sample via the finger prick method to measure high-sensitivity C-reactive protein. At follow-up, participants were interviewed via videoconferencing to ascertain their experiences and feelings related to the study protocol. Qualitative analyses explored the feasibility and acceptability of the study protocol, and exploratory quantitative analyses explored the descriptive statistics and Pearson correlations among the main study variables of interest.

**Results:**

The retention rate was high (19/20, 95%) in our study sample. Qualitative analyses demonstrated that participants were willing to engage in similar, longer-term studies (eg, studies that include both week and weekend days) in the future and suggested the feasibility and acceptability of our study protocol among YSMM. However, participants noted several areas for improvement (eg, redundancy of survey items and difficulty pricking one’s finger) that should be considered in future research. Preliminary quantitative analyses revealed a moderate negative correlation between everyday discrimination and mean cortisol levels (*r*=−0.51; *P*=.03). Furthermore, descriptive analyses suggest that that daily cortisol curves differ across races or ethnicities among YSMM. White and other-identified YSMM experienced the highest cortisol awakening response (mean 0.39, SD 0.21 µg/dL for White participants; mean 0.34, SD 0.34 µg/dL for others) with the steepest decline around bedtime (mean 0.05, SD 0.04 µg/dL for White participants; mean 0.09, SD 0.13 µg/dL for others) followed by a lower cortisol awakening response (mean 0.31, SD 0.11 µg/dL for Hispanic participants; mean 0.23, SD 0.15 µg/dL for Black participants) and a slower decline around bedtime (mean 0.10, SD 0.09 µg/dL for Hispanic participants; mean 0.03, SD 0.02 µg/dL for Black participants) among Hispanic and Black YSMM.

**Conclusions:**

Overall, the results suggest that similar study protocols are feasible and acceptable among YSMM. Future research should highlight the pathways through which cardiovascular disease risk may arise among YSMM using longer-term study designs and more diverse study samples.

## Introduction

### Background

Cardiovascular disease (CVD) is the leading cause of death and disability in the United States [1], and young sexual minority men (YSMM) have disproportionately high rates of CVD compared with their heterosexual counterparts [2]. Emerging evidence implicates stress from racial and sexual orientation discrimination as a significant social determinant of cardiovascular risk among young sexual minorities (aged 18-35 years) [3-5]. Moreover, research shows that CVD risk accumulates early in life, and as such, prevention interventions should focus on addressing risk factors in young adulthood [6]. However, evidence suggests that disparities exist in early CVD risk, such that sexual minorities are more likely to experience CVD at an earlier age than heterosexual individuals, in part because of exposure to minority stress (eg, sexual orientation-related discrimination) [7]. Although research has demonstrated that sexual minority men are more likely to experience excess CVD risk related to factors such as tobacco use, illicit drug use, and poor mental health [8], there is a critical lack of research aimed at understanding the mechanistic links between minority stress and CVD risk, particularly for YSMM. Thus, this study aimed to examine the feasibility and acceptability of an internet-based daily diary protocol aimed at measuring the mechanistic links between minority stress, CVD risk behaviors, and health among YSMM between the ages of 18 and 35 years.

### Theoretical Model

This study was guided by sexual minority stress theory, which posits that sexual minority individuals are exposed to a number of distal stressors related to the negative social valuation of minority identities, resulting in stress exposure beyond the level that people generally experience [9]. Distal minority stressors refer to external discrimination or prejudice events that are targeted toward an individual owing to their minority identity (eg, sexual orientation discrimination) [9]. However, over time, individuals may internalize minority stress, which may, in turn, increase the likelihood of dysregulated physiological stress and poorer health behaviors (eg, substance use) [10-13], which are linked to an increased risk of subclinical CVD [14-17]. Evidence suggests that exposure to sexual minority stressors can lead to physiological changes that over time can lead to an increased risk of CVD [3,18-20]. For instance, Lewis et al [3] posit a causal pathway between discrimination and CVD risk such that exposure to discrimination leads to emotional or affective dysregulation, which, in turn, can lead to physiological dysregulation (eg, inflammation) and poor health coping behaviors (eg, substance use). Over time, this may overburden the physiological stress system and lead to cardiovascular risks. Indeed, these authors found evidence to suggest that expectations of racism are associated with increased carotid intima-media thickening measurements, a measure of increased risk of subclinical CVD, among Black women [3].

Experiences of sexual minority stress among racial or ethnic minorities are not equal and are framed by individual cultural realities and contexts. Intersectionality theory posits that the possession of multiple marginalized identities intertwines at the individual level and is reflective of structural-level power inequities and inequality, influencing health behavior and outcomes across the life course [21]. In particular, the intersection of race- and sexual orientation–based discrimination may be detrimental to health. Indeed, many studies have documented the deleterious effects of discrimination among racial or ethnic minorities, including dysregulated cortisol rhythms, elevated C-reactive protein (CRP), and heightened ambulatory blood pressure [19,22]. These important biological outcomes influence vascular inflammation, atherosclerosis, stroke, and other cardiovascular risk factors [23-25]. In addition, there are health behaviors along the posited causal pathway between discrimination and CVD risk including substance use, diet, and physical activity, which are important mechanisms to study and understand [3,26]. Nevertheless, the mechanisms linking discrimination and cardiovascular factors in racially diverse populations remain underexplored [4]. Conducting this research is vitally important, considering the heightened rates of both stress from discrimination and subclinical CVD among these potentially vulnerable populations [3,8].

The COVID-19 pandemic has provided us with a unique opportunity to conduct our study on the web. Thus, the current investigation reports on the feasibility and acceptability of conducting a study to understand 2 key pathways linking sexual minority and racial or ethnic discrimination to CVD risk, health behaviors (ie, substance use and physical activity), and physiological dysregulation (stress reactivity) completely virtually.

### Study Objectives

The aims of this study were as follows: (1) to assess the feasibility and acceptability of a virtual daily diary study protocol (measured via qualitative debrief interviews), including the collection of biologics and ActiGraph technology; and (2) to explore preliminary associations between discrimination, substance use, sleep, physical activity, physiological stress, and inflammation among YSMM (measured via surveys, ActiGraph technology, and biologics, respectively).

## Methods

### Setting and Participants

This single-site pilot study recruited YSMM between the ages of 18 and 35 years from the New York tristate area. Eligibility criteria included (1) identification as cisgender male; (2) between the ages of 18 and 35 years, inclusive, at the time of screening; (3) identification as a sexual minority; (4) having no known heart conditions, diabetes, or high blood pressure; and (5) willingness to provide informed consent. Participants were identified via online and offline techniques, including posts to listservs and flyers posted on campuses around the New York metropolitan area (eg, New York University). The recruitment materials included information about the study, contact information for the principal investigator’s laboratory, and a link to a web-based screening survey via Qualtrics.

### Ethical Considerations

Interested participants filled out a web-based screening survey, which ascertained relevant sociodemographic information, such as age, biological sex, gender identity, sexual orientation, race, and ethnicity. Eligible participants were contacted by trained research assistants (RAs) to schedule a baseline meeting via Zoom to confirm eligibility, provide more information about the study (eg, study protocol and duration of study), and provide electronic informed consent. The participants were renumerated with up to US $95 for completion of the study protocol. The full details of the full study protocol are detailed below. This study was approved by the Institutional Review Board of New York University (FY2021-5355).

### Procedure

#### Baseline

Participants who were screened eligible through the web-based screening survey were emailed by trained RAs to schedule a baseline meeting via Zoom. During this baseline visit, participants were given detailed information about the full study design, which included a baseline survey to be taken on the web via Qualtrics, a 2-day daily diary period that included biological specimen collection (see 2-Day Diary Period section), and a 30- to 45-minute follow-up and debrief interview via Zoom after completion of the baseline survey and the 2-day protocol. Participants who agreed to participate and who provided electronic informed consent were then sent a link to a Qualtrics survey to be taken within the proceeding 24-hour period as well as electronic copies of step-by-step instructions for saliva collection, HemaSpot collection, and ActiGraph use. Actigraphy was chosen for the purposes of this study because research demonstrates that it provides reliable estimates of sleep patterns in relation to polysomnography [27]. The baseline Zoom meeting took an average of 30-45 minutes, and the baseline survey took approximately 45 minutes to complete. Furthermore, participants who provided informed consent were mailed a study kit with materials needed to complete the 2-day diary period via the United Parcel Service (UPS). The study kit contained saliva collection aids for the 2-day period, a HemaSpot collection device, an ActiGraph watch, and printed copies of instructions for each section of the protocol.

#### Two-Day Diary Period

Within 48 hours of completing the baseline, RAs mailed study kits to participants’ homes via UPS overnight shipping. These study kits contained (1) a charged ActiGraph GT9X Link watch ready to start data collection immediately; (2) 2 Ziploc bags with 3 saliva collection tubes and 3 saliva collection aids per bag; (3) a HemaSpot blood spot collection device with an absorbent sheet, 2 gauze packs, 2 alcohol swabs, 2 safety lancets, and 2 bandages; (4) printed out instructions for the saliva collection procedure, HemaSpot blood spot collection procedure, and wearing the ActiGraph, as well as a sheet to record when the ActiGraph was placed on one’s wrist and when it was taken off, if applicable; (5) supplies for returning the study kit including a small plastic bag with a humidity indicator card and 2 desiccant packets for the HemaSpot device, a cold pack (which they were instructed to freeze immediately upon receiving the kit), a silver insulated bubble mailer, and a brown paper shipping envelope; and (6) a prepaid shipping label to return the ActiGraph, saliva samples, and HemaSpot kits back to the laboratory. Participants were instructed to start the 2-day protocol the day after receiving their study kit.

Over the 2-day study period, participants were instructed to wear the ActiGraph watch on their nondominant wrist consecutively, even when showering and sleeping, over the 2-day period. Furthermore, participants provided 3 saliva samples per day, a blood spot sample, and took 3 surveys each day via Qualtrics. The first 2 of these surveys, termed “momentary diaries” took 2-3 minutes to complete and asked participants to fill out relevant information related to saliva samples, mood, and experiences. The last survey of the day, termed the “nightly diary,” took about 10 to 15 minutes to complete and asked the same “momentary diary” questions as well as more detailed questions about their experiences throughout the day. Surveys were sent via email to participants at 6:00 AM, 6:30 AM, and 8 PM each day over a 2-day period, and they were instructed to take the surveys immediately after providing their saliva samples upon wakeup, 30 to 45 minutes after waking up, and around bedtime.

For the 2-day saliva collection period, participants were asked to provide 3 saliva samples per day via the passive drool method. The first saliva sample was taken immediately upon waking, the second saliva sample was taken 30-45 minutes after waking, and the third saliva sample was taken before bedtime. This procedure was repeated for 2 consecutive days. Participants were instructed to avoid brushing their teeth, eating, and drinking liquids high in sugar or acidity (eg, coffee) 30 minutes before taking their saliva samples. Participants were instructed to allow saliva to pool in their mouth and then use a saliva collection aid to collect 1 mL of saliva in the collection vial. The participants were also instructed to freeze their samples immediately after collection.

**Figure 1:**
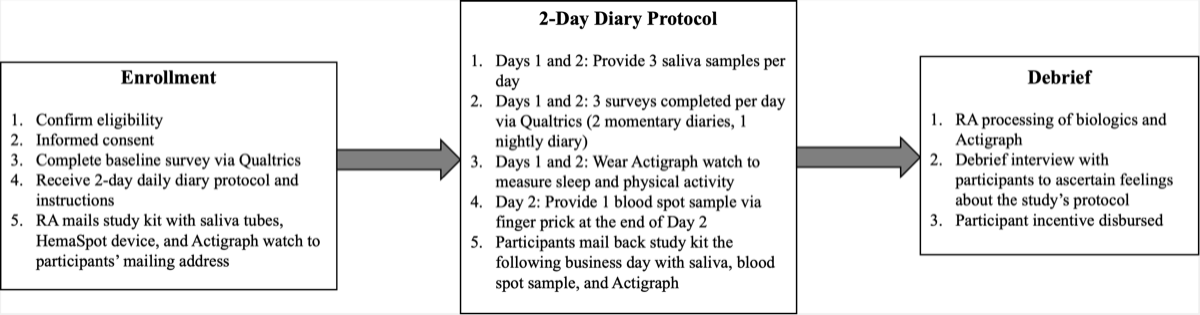
Overview of the study procedures. RA: research assistant.

For the HemaSpot blood spot collection procedure, participants were instructed to provide a blood spot sample on the afternoon of the second day of the 2-day study period. Participants were instructed to wash their hands before beginning the finger prick procedure. Then, participants spread the absorbent sheet provided in the study kit on a table or countertop and placed gauze, alcohol swabs, safety lancets, and bandages on the sheet in close reach. When the participants were ready to start the procedure, they were instructed to open the pouch containing the HemaSpot device (which must be used within a few minutes of opening). They were then instructed to prepare a safety lancet for use by twisting the cap until it popped out. Participants then selected their ring or middle finger from their nondominant hand and selected a place to the side of the finger pad (avoiding their fingernail). Participants then wiped their finger with an alcohol swab, allowing it to air dry, and then placed their hand palm up on the absorbent sheet and pressed the safety lancet firmly against the site until it clicked. They were then instructed to squeeze gently to produce a drop of blood, wipe the first drop away with gauze, and then begin collecting with the second drop. Participants then positioned their finger over the HemaSpot device funnel and were instructed to collect 3-4 drops of blood (and to avoid oversaturation). If they were not able to obtain enough blood on the first attempt, they were told they were able to try again with the second safety lancet. They were then instructed to use the gauze pad to wipe away extra blood and to apply a bandage to the site immediately thereafter. Finally, the participants were told to close the HemaSpot device and wait 3-4 hours for the blood to completely soak into the paper and dry on a flat surface. After this waiting period, the participants were instructed to freeze their blood spot samples before mailing their study kit back. Figure 1 presents an overview of the study’s procedure.

#### Financial Incentive Structure

In this study, a financial incentive structure was used to promote retention. Participants were compensated with US $10 for completion of the baseline survey. For wearing the ActiGraph and returning the ActiGraph, participants were compensated with US $15. To provide and return saliva samples, the participants were compensated with US $15. For providing and returning the blood spot sample, participants were compensated with US $15. Furthermore, the participants were provided with US $10 for completing the follow-up interview. Finally, if participants completed each component of the study, they received a US $30 bonus. Thus, the participants could earn up to US $95 for the completion of this study.

### Qualitative Data

#### Procedure

Participants were instructed to mail their study kits to the laboratory the day after completing the 2-day study protocol by placing the frozen HemaSpot device in a small plastic bag with a humidity indicator card and the 2 desiccant packets. They were instructed to place the 6 saliva collection tubes and ActiGraph along with the sealed HemaSpot device in a silver-insulated bubble mailer with a frozen cold pack. Afterward, participants placed the silver-insulated bubble mailer into the brown paper silver envelope and were instructed to affix the prepaid return label to the envelope and drop it off at UPS to be sent back to the laboratory via overnight shipping.

After receiving the returned study kit back to the laboratory, RAs processed the saliva and blood spot samples by examining the features of the saliva (eg, denoting whether phlegm or blood was present) and blood spot samples (eg, if there was too much or too little blood). Furthermore, ActiGraph data were downloaded. Then, the RAs contacted the participants via email to schedule their 15 to 20 minute debrief interviews via Zoom. During this interview, the RAs described the purpose of the study and followed a semistructured interview guide to ascertain participants’ experiences with the study design, collecting saliva and blood spot samples, wearing an ActiGraph, and filling out diary surveys each day. After the completion of this interview, participants were compensated according to their compliance with the study protocol (see the Financial Incentive Structure section above) through an electronic Visa gift card via GiftBit.

The RAs took notes on participants’ responses to the follow-up interview questions (see Figure 1). The interview guide was structured to gather information pertaining to the feasibility and acceptability of the study protocol among the YSMM. In particular, we asked participants about their experiences with the RAs, saliva samples, ActiGraph watch, and HemaSpot collection device. We also asked participants to describe whether the study caused any stress or confusion, and if they had any areas of improvement to increase their willingness to engage in future research with similar designs.

#### Qualitative Analysis Plan to Assess Feasibility and Acceptability

A deductive directed content analysis approach was used to code the interview notes [28]. This approach uses prior theory and research to create an initial coding scheme consisting of overarching themes—in this case, our initial predetermined themes were feasibility and acceptability as well as potential areas of improvement. Three coders met to discuss the predetermined themes and then one coder applied the themes to the transcripts. The 3 coders met throughout the duration of the qualitative analysis to discuss themes and resolve any discrepancies in coding. After reading the transcripts, subthemes for each overarching theme were deduced from the interview notes to determine a finalized coding scheme organized by the themes of feasibility, acceptability, and areas for improvement. Debrief notes were analyzed using Atlas.ti qualitative software [29].

### Quantitative Data to Explore Preliminary Associations

#### Baseline Survey

The baseline survey was conducted on the web using Qualtrics. Participants were assigned a unique participant ID and required to enter the ID to access each survey. The baseline survey asked questions pertaining to sociodemographics (eg, date of birth, race and ethnicity, education level, yearly income), family history of cardiovascular-related illness or disease, and general weekly exercise patterns (ie, the Global Physical Activity Questionnaire [30]). We then administered a series of scales related to experiences with stress (eg, Adverse Childhood Experiences Scale [31], Perceived Stress Scale [32]), discrimination (Everyday Discrimination Scale [EDS] [33], Major Experiences of Discrimination Scale [33]), substance use (Alcohol, Smoking, and Substance Use Involvement Screening [34]), and mental health (eg, Center for Epidemiologic Studies Depression Scale [35]). The baseline survey took an average of 45 minutes to an hour to complete.

#### Momentary Diaries

Momentary diaries were taken by participants twice per day over a 2-day period (once upon waking and once 30-45 min after waking) via Qualtrics. Participants were asked several questions related to their saliva samples (eg, what time they provided the sample and caffeine consumption) and questions pertaining to their current mood, stress, recent experiences of racial and sexual orientation discrimination, and substance use. Each momentary survey was sent to the participants via a secure Qualtrics link. The survey took an average of 2-3 minutes to complete.

#### Nightly Diaries

Nightly diaries were taken by participants once per day over a 2-day period (before bedtime) using Qualtrics. Nightly diaries contained the same questions as the momentary diary and included modified scales to assess experiences of mood (Positive and Negative Affect Schedule [36]), depressive symptoms (Center for Epidemiologic Studies Depression Scale), and discrimination (EDS). Nightly diaries were sent to the participants via Qualtrics via email.

#### Salivary Cortisol

Participants provided 3 saliva samples per day over the course of 2 consecutive days to assess diurnal cortisol [37]. They were instructed to provide samples upon awakening, 30 minutes after awakening, around lunchtime, and around bedtime. The participants were also instructed to take a brief diary survey after providing each saliva sample. After participants collected their first 3 saliva samples, they received a link to an additional “momentary” diary survey via text message or email, which took about 2 minutes to complete. This momentary survey assessed variables associated with cortisol (eg, caffeine consumption) as well as other psychosocial variables (eg, current mood). Finally, after each bedtime saliva sample, participants received a link via text message or email, depending on their preference, to a longer “nightly” diary survey. To increase adherence to sample collection and surveys, participants received text message reminders to provide their samples, which also contained the appropriate survey link.

#### High-Sensitivity CRP

The Center for Studies in Demography and Ecology Biodemography Lab analyzed serum high-sensitivity CRP (hsCRP) values using an enzyme-linked immunosorbent assay as described in detail elsewhere [38]. Microliter plates were coated with anti-CRP antibodies to measure CRP concentrations within serum samples and stored at −20 °C. This method has been validated for population health research as a robust method for detecting low concentrations of CRP [38-40].

hsCRP values were arranged into 3 categories according to clinical risk for CVD assigned by the American Heart Association and Centers for Disease Control and Prevention to create a nominal outcome variable for calculation: low (<1.00 mg/L), average (1.00-3.00 mg/L), and high (>3.00 mg/L) [41,42]. The categories described the results in terms of clinical significance, as CVD risk increases with higher CRP concentrations. Previous studies using CRP in heart disease have demonstrated this clinical significance using categorical CVD risk [43-45].

### Other Covariates

#### Overview

BMI was calculated using a standard formula based on the participants’ height and weight (kg/m^2^), each of which was self-reported. Values were sorted into 2 categories: underweight or normal weight (<25.00) and overweight or obese (≥25.00). Furthermore, we also ascertained information pertaining to smoking status (never, former, and current), substance use (measured at baseline via Alcohol, Smoking, and Substance Use Involvement Screening and nightly diaries), and family history of CVD (yes or no).

#### Quantitative Analysis

First, sociodemographic and clinical characteristics were explored in our study sample (eg, means with SDs and frequencies). We then explored retention and feasibility through an examination of study dropout, loss to follow-up, and completion rates of each of the study components (ie, salivary samples, HemaSpot blood spot samples, momentary diaries, and nightly diaries). Finally, Pearson correlation coefficients were used to explore associations among our main independent variables (discrimination as measured by EDS), the dependent variable (hsCRP), and our mediators (sleep, physical activity, and physiological stress as measured by salivary cortisol). Time-varying variables (ie, sleep, physical activity, and cortisol) were averaged across the study period for each participant. Baseline discrimination (as measured by EDS) was used for the purpose of the correlations. Average daily cortisol curves were also examined and stratified by race and ethnicity using the profile plot command in Stata (version 18; StataCorp) [46].

## Results

### Sample Characteristics

**Figure 2:**
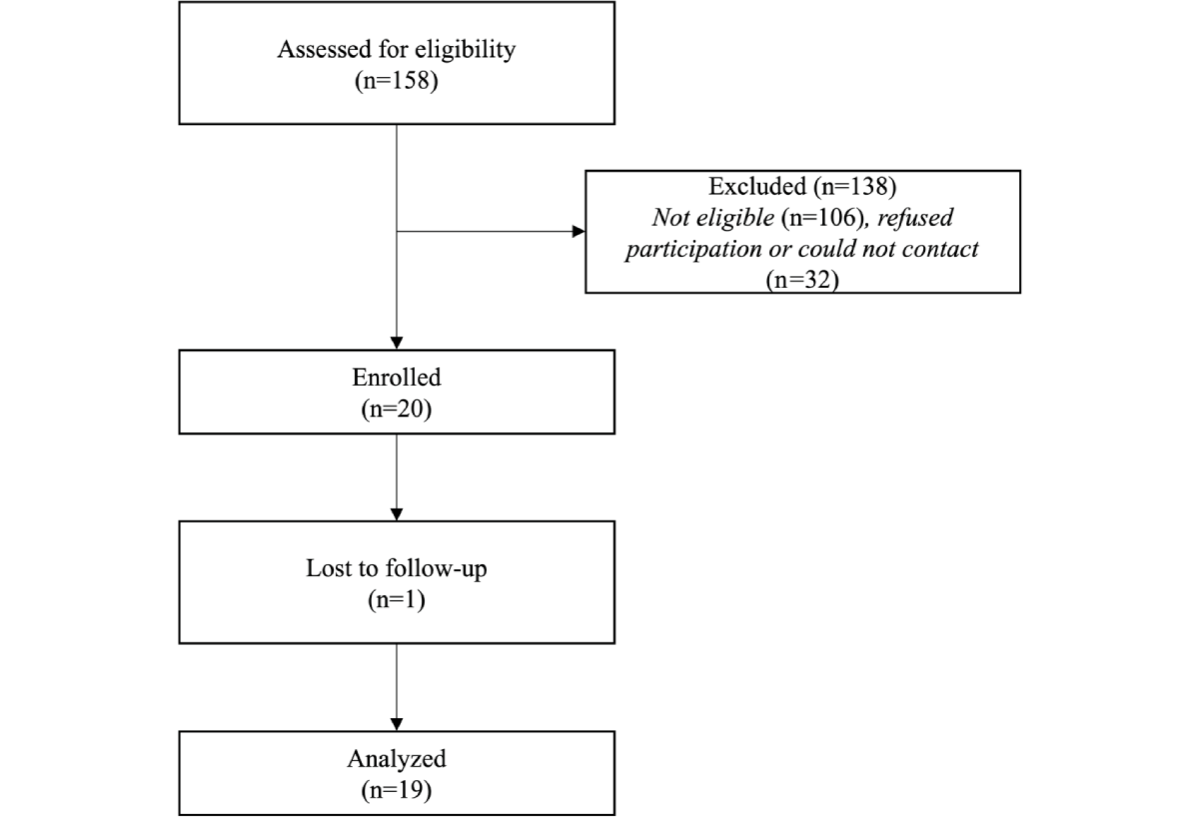
Participants screened, excluded, enrolled, and analyzed.

Figure 2 displays an overview of the number of participants who were screened, ineligible, enrolled, and analyzed. Sociodemographic and clinical characteristics of the 19 YSMM who participated in the study are presented in Table 1. On average, the participants were aged 24.37 (SD 5.46) years. With respect to racial or ethnic identity, 39% (7/19) identified as non-Hispanic white, whereas the remaining identified as Hispanic (3/19, 16%), Black (3/19, 16%), or another racial or ethnic identity. Most participants identified as gay or bisexual (8/19, 42%), and the remaining identified as having another sexual identity. Concerning health characteristics, most participants reported an average BMI (10/19, 53%) and no family history of CVD (16/19, 84%). The average hsCRP among participants was 0.20 (SD 0.17), and participants slept an average of 313.18 (SD 90.25) minutes per night and expended an average of 1.52 (SD 0.36) metabolic equivalent of tasks (METs) per day.

Table 2 presents the retention and feasibility measures. Overall, 20 YSMM enrolled in this study. Of these 20 YSMM, 1 participant was lost to follow-up after the informed consent meeting, and 2 participants were lost to follow-up after completion of the study protocol (ie, they did not participate in the follow-up interviews but completed all other parts of the study protocol). Of the 19 YSMM who engaged in the study protocol, 100% (n=19) sent back their HemaSpot samples (ie, blood spots) and all 6 of their saliva samples (3 saliva samples per day for 2 consecutive days). With respect to the momentary diaries, 89% (17/19) completed 4 out of the 4 momentary diaries (2 momentary diaries/d for 2 days). The remaining participants completed either 2 (5%; n=1) or 1 (5%; n=1) of the momentary diaries over the 2-day period. In contrast, 1 participant did not complete any nightly diaries over the 2-day period. However, of the remaining 18 participants, 94% (n=16) completed 2 out of the 2 nightly diaries over the study period, and the remaining 6% (n=2) completed 1.

**Table 1:** Sociodemographic and clinical characteristics of the study sample (n=19).

Characteristics		Value
Age (y), mean (SD; range)		24.37 (5.46; 18-35)
**Race or ethnicity^a^, n (%)**		
	White, non-Hispanic	7 (39)
	Hispanic	3 (17)
	Black, including Hispanic	3 (17)
	Other	5 (28)
**Sexual orientation, n (%)**		
	Gay	8 (42)
	Bisexual	8 (42)
	Other	3 (16)
**Education, n (%)**		
	High-school diploma or GED^b^	4 (21)
	Some college	8 (42)
	Graduate degree	7 (37)
**BMI category, n (%)**		
	Normal (<25)	10 (53)
	Overweight or obesity (³25)	9 (47)
**Family history of CVD^c^**		
	Yes, n (%)	3 (16)
	No, n (%)	16 (84)
	hsCRP^d^, mean (SD; range)	0.20 (0.17; 0.03-0.63)
	Number of min of sleep/night^e^, mean (SD; range)	313.18 (90.25; 181-487)
	METs^f^/d, mean (SD; range)	1.52 (0.36; 1.09-2.73)
**Cortisol measures (µg/dL), mean (SD)**		
	Upon waking	0.33 (0.15)
	30-45 min after waking (CAR^g^)	0.33 (0.22)
	Around bedtime	0.06 (0.08)

^a^One respondent had missing data regarding race.

^b^GED: General Educational Development.

^c^CVD: cardiovascular disease.

^d^hsCRP: high-sensitivity C-reactive protein.

^e^Two participants had missing actigraphy data on sleep.

^f^MET: metabolic equivalent of task.

^g^CAR: cortisol awakening response.

**Table 2:** Retention and feasibility of the study protocol (n=19^a^).

		Values
HemaSpot samples returned, n (%)		19 (100)
Saliva samples returned, n (%)		114 (100)
Momentary diaries completed, mean (SD; range)		2.45 (1.13; 1-4)
Nightly diaries completed, mean (SD; range)		1.47 (0.51; 1-2)
**Momentary diary completion by day, mean (SD)**		
	Average diaries completed; day 1 (n=19), mean (SD; range)	1.49 (0.51; 1-2)
	Average diaries completed; day 2 (n=17), mean (SD; range)	1.50 (0.51; 1-2)
**Nightly diary completion by day, n (%)**		
	Sample completed, day 1	18 (95)
	Sample completed, day 2	16 (84)

^a^20 participants enrolled in the study; however, only 19 (95%) completed the protocol.

### Feasibility and Acceptability

Overall, 17 (89%) participants completed the follow-up qualitative interviews. Within each overarching prespecified theme of “feasibility,” “acceptability,” and “areas for improvement,” we found several subthemes. The overarching themes and subthemes, as well as their definitions and excerpts, are presented in Table 3. For feasibility, we found two subthemes: (1) informative onboarding experience and easy communication with RAs, and (2) easy protocol to follow. Most participants (14/17, 82%) reported that their onboarding experience (including informed consent and a detailed step-by-step protocol) was informative and that they were able to answer all of their questions. Several participants noted that the documents that were emailed (and hard copies mailed) to them during the onboarding call were clear and laid out the study protocol in a helpful and step-by-step manner. Furthermore, participants noted that the RAs were easy to communicate with and were available to answer any questions they may have had over the study protocol period, thus reducing any confusion or hesitancy when it came to completing the study’s steps. Furthermore, 76% (13/17) reported that the study protocol was easy to follow with little to no issues arising, especially with the instruction documents that were sent to the participant (eg, for the ActiGraph, HemaSpot, and saliva samples).

With respect to acceptability, we found three subthemes: (1) appropriate financial incentive structure, (2) desire to participate beyond financial incentives, (3) changed perceptions of mood or discrimination, and (4) willingness to participate in long-term studies with a similar protocol. First, 53% (9/17) reported that the financial incentive payment structure increased their willingness to complete the study and all steps in the protocol. These participants noted the importance of being compensated fairly for their time, especially because this protocol required the collection of biological samples (ie, saliva and blood). Furthermore, these participants also noted that they appreciated the breakdown of the payments—in other words, they appreciated being compensated for each component of the study separately rather than a lump contingent on completion of the entire protocol at the end of the study period. However, 41% (7/17) of the participants described internal and altruistic motivations for participation, which motivated them to complete the study regardless of the financial incentives attached to it. These participants described an interest in the scientific process, as well as wanting to contribute to their community’s health by getting involved in health-related research. Moreover, 47% (8/17) reported positive experiences directly because of participation, including a better sense of mood and becoming more aware of experiences of discrimination. These participants noted that reflecting on their experiences over the course of the day (eg, nightly diaries) required them to become more mindful of their feelings and moods throughout the day and to become more aware of their surroundings. Furthermore, these individuals were able to better capture experiences with discrimination and microaggressions and reflect on their subsequent moods. Finally, 100% (n=17) of the participants reported willingness to participate in long-term studies (eg, 30 days of data collection) using a similar protocol. In particular, participants felt that a long-term study would be more reflective of their day-to-day stressors and experiences. However, 2 of these participants noted that while they would be willing to participate in a longer-term study, they preferred that the study surveys (eg, baseline and nightly) be shorter in duration.

**Table 3:** Qualitative themes and subthemes (n=17^a^).

Theme and description				Example quotes		Endorsed, n (%)
**Feasibility of intervention**						
	Informative onboarding experience and easy communication process with RAs	Descriptions related to the ease of understanding the onboarding instructions and documents and being able to communicate in a timely manner with the RAs as well as get their answers answered promptly	“Yes, [the onboarding experience] answered all the questions at that point and I had a few more questions [later] but I was able to get my questions answered.” (Hispanic, bisexual, 28 y) “Everything was fine, [it was] very pleasant to talk with research assistants. Instructions were clear granted how much information there was.” (White, gay, 35 y)		14 (82)	
	Protocol easy to follow	Participant was able to comprehend and follow the study instructions with little to no problems	“[The protocol was a] good experience, not very invasive. Labeled tubes and collection aid were helpful. Instructions were clear and helped.” (Asian, bisexual, 21 y) “Everything was very clear. Explanation of the blood spot sample was especially helpful.” (Asian, gay, 28 y)		13 (76)	
**Acceptability of intervention**						
	Appropriate financial incentive structure	Descriptions of being motivated to engage in the study due to the financial incentive structure and feeling that the financial incentive structure was appropriate for the time given	“The incentive did help my willingness [to participate] and the breakup of the payments helped.” (Hispanic, bisexual, 25) “[The financial incentive structure] made me want to ensure I completed all the steps, but it was not very hard so I would have done it either way. But the payments definitely helped.” (White, bisexual, 30 y)		9 (53)	
	Desire to participate beyond financial incentives	Feelings and motivations of external reasons for participating in the study (eg, wanting to progress science)	“From an academic perspective, I was interested in the study regardless. Realistically, I would be taking opportunity to be doing the study anyway.” (Black Hispanic, gay, 31 y) “[The financial incentive structure] did not really affect my participation, would have still done without compensation.” (Black, gay, 31 y)		7 (41)	
	Changed perceptions of mood and discrimination	Increased awareness of one’s experiences with discrimination and mood throughout the day	“It made me a bit more aware of my surroundings and did notice more [discriminatory] experiences once I was asked.” (Hispanic, gay, 18 y) “[I became] more sensitive to attuning to [discrimination]. Many of these things I encounter on a regular basis, but it became more salient when calling my attention to it.” (Black Hispanic, gay, 31 y)		8 (47)	
	Willingness to participate in longer-terms studies	Expressing interest and desire to participate in studies similar to this on a longer-term basis (eg, 30 d)	“Yeah, it would be good to participate [in a longer term study] because I felt like study duration was very short. A longer study would be more insightful.” (Black Hispanic, gay, 24 y) “Absolutely [I would participate in a longer-term study]. I have participated in longitudinal studies before and recognize the challenge that goes into them.” (Black Hispanic, gay, 31 y)		17 (100)	
**Areas for improvement**						
	Day-to-day stressors unrelated to study	Daily stress that may have interfered with participants’ abilities to adhere to protocol	“No stress related to the study. Logistical planning was needed to make sure I was waking up and going to bed at a relatively set time. But it was not stressful, just extra planning in terms of how I would navigate my time.” (Black Hispanic, gay, 31 y) “I stressed during the 2-day study due to personal relationships. The study didn’t cause any anxiety, and I thought the structure was helpful and allowed me to plan things out.” (Asian, bisexual, 21)		9 (53)	
	Protocol-related criticism	Criticism related to protocol-related activities (eg, survey items, saliva procedure)	“The surveys were long and felt frustrating. I felt the wording was ambiguous and that the questions assumed participants were affected by other people’s feelings.” (White, bisexual, 21 y) “It was a good experience during the day, but at night after doing the blood sample it was a little bit exhausting and it was a lot of questions, etc.” (Hispanic, gay, 18 y)		13 (76)	
	Stress directly resulting from study protocol	Stress stemming from participating in the study	“Certain parts of the study caused me stress, such as the blood spot sample.” (Asian, bisexual, 19 y) “To be honest, it was a [stressful] experience, it was simple to give saliva, etc. but I was uncomfortable with storing the saliva in the freezer. It was hard to use the saliva tube and straw, but at the end of the day it was a good experience. It was also hard to write the time on the tube.” (Black Hispanic, gay, 24 y)		4 (24)	

^a^Of the 19 participants who completed the protocol, 2 did not complete follow-up interviews (17/19 completed follow-up interviews).

Although participants overall had positive feedback with respect to the study design and their experiences with participation, there were several areas that they noted for improvement: (1) experiences with day-to-day stress unrelated to the study, (2) protocol-related criticisms related to the study procedures, and (3) stress directly resulting from the study protocol. First, 53% (9/17) of the participants noted that they dealt with various forms of daily stress unrelated to the study protocol (eg, relationship stress and school stress). Due to these various daily stressors, some participants noted that they had to take extra time to plan ahead and ensure that they were able to complete the steps of the study in the specified time frame. While not inhibiting their ability to complete the study, they noted that planning took extra time and mental energy. Second, 76% (13/17) of the participants specified criticism that was directly related to the study protocol. In particular, 9 of these participants noted frustration with the survey instruments. These participants noted that the surveys, particularly the nightly diary, were long and repetitive. Furthermore, some participants also noted the need to be more inclusive of other identities (eg, trans, nonbinary), although this was beyond the scope of this study. Moreover, 6 of these participants noted that it was difficult to collect their blood spot samples and had difficulty producing enough blood for the HemaSpot collection device, although they also noted that the inclusion of 2 lancets was helpful. Finally, a few (4/17, 24%) participants noted that they experienced stress stemming directly from engaging in the study. In particular, participants noted stress when having to prick their finger for a blood spot sample (eg, needle anxiety) and having to produce saliva samples 3 times a day. Furthermore, one participant noted that they were not comfortable storing their saliva samples in freezers until they were ready to ship back to the laboratory.

### Preliminary Exploration of Hypothesized Pathway

**Figure 3:**
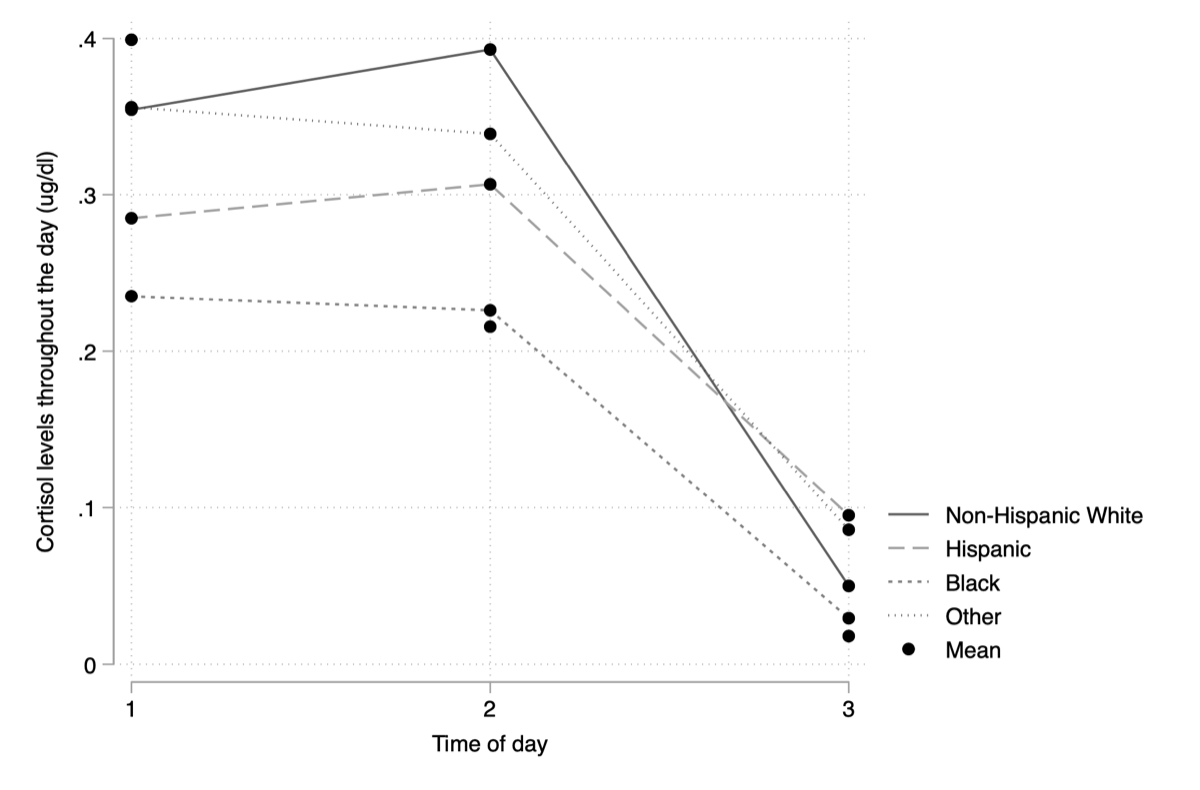
Average daily cortisol curves by racial or ethnic identity; 1, 2, and 3 on the x-axis refer to the first sample of the day (upon awakening), the second sample of the day (+30 min after waking), and the third sample of the day (around bedtime), respectively.

Correlations between main independent variable (discrimination), dependent variable (hsCRP), and the mediators (salivary cortisol, sleep, and physical activity) are presented in Table 4. Furthermore, Table 5 displays the average cortisol parameters by race, and Figure 3 displays the average cortisol curves over the 2-day period stratified by race or ethnicity. It is important to note that out of the 19 YSMM included in these analyses, 2 participants did not wear their ActiGraph watch over the 2-day period and thus were not included in the actigraphy measures. There was a moderate negative association between everyday discrimination and mean cortisol such that greater experiences of discrimination were associated with lower daily cortisol, on average. Moreover, we also observed a moderate, positive association between daily physical activity, on average (as measured by METs) and hsCRP such that greater levels of average physical activity were associated with higher hsCRP, on average. No other correlations were significant in our study sample. With respect to the average cortisol curves over the 2-day period (Figure 3), White YSMM experienced the strongest cortisol awakening response (CAR; ie, the spike in cortisol that occurs in healthy individuals 30-45 min after waking), which steeply declined throughout the day, whereas Black YSMM’s cortisol started out lower and exhibited a weaker CAR and declined throughout the day.

**Table 4:** Correlation matrix (n=19^a^).

	Everyday discrimination	Mean cortisol (µg/dL)	Mean sleep (min)	Mean METs^b^	hsCRP^c^ (mg/L)
Everyday discrimination	—^d^	—	—	—	—
Mean cortisol (µg/dL)	−0.51^e^	—	—	—	—
Mean sleep (min)	−0.07	−0.14	—	—	—
Mean METs^f^	−0.32	0.31	−0.16	—	—
hsCRP (mg/L)	0.04	0.29	−0.18	0.59^g^	—

^a^Two participants did not complete follow-up interviews.

^b^MET: metabolic equivalent of task.

^c^hsCRP: high-sensitivity C-reactive protein.

^d^Not applicable.

^e^*P*=.03.

^f^MET: metabolic equivalent of task.

^g^*P*=.008.

**Table 5:** Cortisol by race (n=19^a^).

	White (n=3), mean (SD)	Hispanic (n=3), mean (SD)	Black (n=7), mean (SD)	Other (n=5), mean (SD)
Waking cortisol (µg/dL), mean (SD)	0.35 (0.18)	0.28 (0.05)	0.24 (0.04)	0.36 (0.19)
Cortisol awakening response (µg/dL), mean (SD)	0.39 (0.21)	0.31 (0.11)	0.23 (0.15)	0.34 (0.34)
Bedtime cortisol (µg/dL), mean (SD)	0.05 (0.04)	0.10 (0.09)	0.03 (0.02)	0.09 (0.13)

^a^Correlations for sleep include 17 out of 19 participants.

## Discussion

### Principal Findings

This pilot study found evidence for the feasibility and acceptability of a virtual daily diary protocol and explored preliminary associations between discrimination, cardiometabolic health behaviors, and inflammation among YSMM. Of the 19 participants who completed more than just the baseline survey, 100% (n=19) returned their HemaSpot and saliva samples, and 89% (n=17) completed all momentary measures.

Although some participants mentioned some areas for improvement, such as having to take extra time to plan ahead to ensure that they were able to complete the required steps and experiencing stress directly related to the study (eg, needle anxiety, having to produce saliva samples 3 times a day), 100% of the participants reported a willingness to participate in longer-term studies using a similar protocol. Such valuable findings greatly contribute to the literature by demonstrating the feasibility and acceptability of a fully virtual protocol with a component of collecting biological specimens. Our findings expand on current research that highlights the specific challenges of conducting a fully virtual protocol [47,48].

For instance, Feigelson et al [47] examined the feasibility of an at-home study involving cross-sectional survey data and stool collection and found that there is a need for significant personal contact and carefully timed follow-up to ensure participant willingness. In our study, only 2 individuals did not participate in follow-up interviews. However, they completed all the other parts of the study protocol. In addition, the subthemes found in the qualitative analysis regarded the informative nature of the onboarding experience, easy communication process with the RAs, and the fact that the protocol was easy to follow. Furthermore, some of the challenges encountered by the participants were concurrent with previous research that has described remote protocols. For instance, in-home sample collection and the inconvenience of fitting the protocol timeframe with participant schedules have been highlighted in previous studies [47,48]. In the current protocol, some of the participants mentioned that due to various forms of daily stress unrelated to the study, they had to dedicate time to ensure they would be able to complete the steps in the specified time frame. In addition, although 76% (13/17) of the participants reported that the study protocol was easy to follow, 24% (4/17) reported experiencing stress when collecting biospecimens. It was not clear if the experiences of stress outside the study protocol would still be a barrier if participants were to come to the laboratory to complete the study.

Furthermore, a notable finding of our protocol was that 47% (8/17) of participants reported better perceived mood and awareness of discriminatory experiences as a direct result of their participation in the study. The participants highlighted that the momentary diary prompts led them to reflect on their experiences throughout the day. As previously described in the study’s theoretical model, there is a pressing need for studies to take an intersectional approach and consider the unique experiences of stress among sexual minority individuals by racial/ethnic and sexual/gender status and by culture and context. Although not a direct aim of our protocol, such results and reports greatly contribute to and expand on the current literature by highlighting the need for protocols and intervention development to take an intersectional approach, especially ones using ecologic momentary data collection. In addition, as mentioned above, 100% of the participants reported a willingness to participate in a longer-duration protocol with components similar to the current one. This was because participants believed that a longer-term study would better reflect their everyday stressors and experiences. Future research aimed at understanding key minority stressors and their effects on health behaviors should consider expanding the study duration to best capture individuals’ overall experiences. Similar study designs with longer durations have shown acceptance among sexual minority populations [49]. For example, one ecologic momentary assessment study examining tobacco, alcohol, and drug use in relation to daily discrimination experiences among 50 sexual and gender minorities found that participants completed an average of 68% of the 6 prompts sent to them daily over a period of 14 days [49]. Furthermore, in a daily diary study examining minority stress and daily mood among racially diverse sexual minority youth (n=94) over a 21-day period, participants responded to 83% of the daily diary prompts sent to them per day [50]. By capturing day-to-day experiences over a longer duration (eg, a 14- or 30-day period), researchers could not only improve generalizability and power but also capture variation in experiences of minority stress and cardiovascular risk behaviors across both week and weekend days.

Our preliminary descriptive findings also shed light on potential differences across racial or ethnic groups in the YSMM, which necessitates further exploration. For instance, we examined average daily cortisol curves across race or ethnicity and found evidence suggesting that Black YSMM may experience a more dysregulated cortisol curve compared with White YSMM. In particular, we found that Black YSMM experienced a less pronounced CAR and a slower decline throughout the day compared with White YSMM. CAR represents a brief period, typically 30 to 45 minutes after waking, where an individual experiences increased cortisol activity and is recognized as an important marker of the hypothalamic-pituitary-adrenal axis as it aids an individual in their transition from sleep to wake [51]. Typically, however, individuals experience a steady decline in cortisol activity throughout the day, with the lowest levels recorded around bedtime (ie, daily cortisol curves) [52]. Research has generally found that individuals with steeper cortisol curves (ie, cortisol that declines at a faster rate) are associated with better overall health outcomes compared with individuals with more blunted cortisol curves (ie, cortisol that declines at a slower rate) [52]. Although we were underpowered to detect significant differences within daily cortisol curves among our study sample (n=19), our descriptive findings shed light on potential differences across race or ethnicity among YSMM that warrant further exploration with larger, more diverse study samples.

Although our preliminary study highlights the feasibility and acceptability of a remote protocol examining momentary, daily, and biological measures, there are several limitations that should be considered. First, our sample consisted of YSMM who resided in the New York tristate area, and thus may not be generalizable to the wider population of YSMM in the United States. Second, our study sample was small, which also affected our ability to detect significant differences in our key measures of interest (eg, daily cortisol curves and CRP). To the best of our knowledge, this is the first study to integrate momentary assessment data and biologics using a fully remote protocol. Third, our study was, in part, disrupted by the COVID-19 lockdown, which may have impacted participants’ ability to fully engage with the protocol and may also have impacted participants’ daily cardiovascular health behavior patterns (eg, participants may not be walking or traveling as much as they used to prepandemic). Fourth, we may have missed important information within our surveys that could affect cardiovascular health, such as dietary patterns. Fifth, although the overarching literature supports the use of wearables, such as the ActiGraph, to measure sleep, it may not have wholly captured sleep patterns over the course of 2 days in comparison with gold standards, such as polysomnography [27,53]. Thus, it is important to carefully examine the updated literature when determining the best wearable device for capturing sleep patterns. Sixth, given that our pilot study only spanned a period of 2 days, our actigraphy measures (ie, sleep and physical activity) may not have reliably captured daily sleep and physical activity patterns among our sample of YSMM and we did not include data on napping in these analyses. Thus, future research should use longer collection periods to capture sleep and physical activity patterns more comprehensively among diverse samples of YSMM. For instance, research has found that a period of at least 3-5 days is necessary to capture accurate information pertaining to sleep [27]. Despite these limitations, the strengths of this study lie in its diverse sample of YSMM and its rigorous design that integrates surveys and biological metrics.

By demonstrating the feasibility and acceptability of a fully remote protocol combining ecological momentary assessment and biological measures, our results pave the way for future work aimed at developing culturally tailored just-in-time interventions that might be delivered virtually. Such interventions should consider minority stressors and their influence on health behaviors and adverse outcomes throughout their lifespan. Doing so could lessen the mental and physical burden at the intersection of multiple minoritized identities as well as reduce inequities in the health care system.

### Conclusions

Overall, the fully virtual protocol for assessing CVD among emerging adult sexual minorities was acceptable, indicating that it can be used in the future for similar work and with a larger time period and sample size. Furthermore, preliminary descriptive results suggest that there may be key racial or ethnic differences among YSMM in key cardiovascular health features (ie, daily cortisol curves) that warrant further research.

## Abbreviations

CAR: cortisol awakening response
CRP: C-reactive protein
CVD: cardiovascular disease
EDS: Everyday Discrimination Scale
hsCRP: high-sensitivity C-reactive protein
MET: metabolic equivalent of task
RA: research assistant
UPS: United Parcel Service
YSMM: young sexual minority men
